# Author Correction: Time-dependent memory transformation along the hippocampal anterior–posterior axis

**DOI:** 10.1038/s41467-018-04516-x

**Published:** 2018-05-24

**Authors:** Lisa C. Dandolo, Lars Schwabe

**Affiliations:** Department of Cognitive Psychology, University of Hamburg, 20146 Hamburg, Germany

Correction to: *Nature Communications* 10.1038/s41467-018-03661-7, published online 23 March 2018

In the originally published version of this Article, the rightmost graph in Fig. 2c was inadvertently replaced with a duplicate of the central panel. This has now been corrected in both the PDF and HTML versions of the Article.

