# Supplementary material for: Time-dependent memory transformation along the hippocampal anterior–posterior axis
Source: Nat Commun. 2018 Mar 23;9:1205. doi: 10.1038/s41467-018-03661-7 (PMC5865114; doi:10.1038/s41467-018-03661-7)
Supplement: Supplementary file 1 — Supplementary Information (PDF 1823 kb) [file 41467_2018_3661_MOESM1_ESM.pdf]

Supplementary Information

Time-dependent memory transformation along the hippocampal anterior-posterior axis

Dandolo & Schwabe

Department of Cognitive Psychology, University of Hamburg, 20146 Hamburg, Germany

Supplementary Figures 1-9

Supplementary Tables 1-3

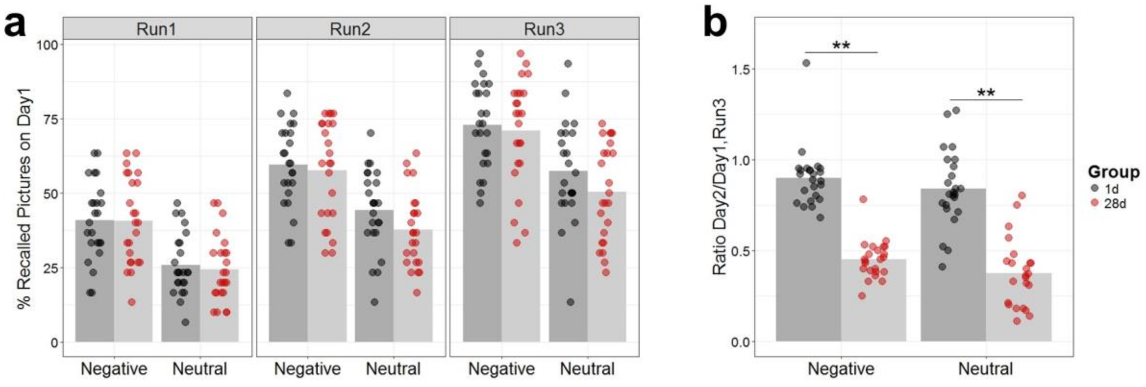

Supplementary Figure 1: Free Recall Performance. (a) On Experimental Day 1, participants of both groups learned equally well with an increase over the three encoding runs. At the end of the encoding session, participants recalled on average 62.88% of the pictures correctly (SEM = 1.90, Run3). Overall, negative pictures (mean = 71.88%, SEM = 2.32, Run3) were better remembered than neutral ones (mean = 53.89%, SEM = 2.40, Run3; paired t-test: t(47) = 8.91, p = 1.16e-11, Cohen's d = 1.29, n = 48). **(b)** For the Free Recall results from Day 2 we looked at a ratio (Day2/Day1-Run3) comparing the results from Day 2 in relation to the encoding performance on Day 1 (Run3), in order to look at differences in the free recall performance independent of initial learning performance. Participants in the 28 d-group (mean = 0.411, SEM = 0.023) recalled significantly fewer pictures than participants of the 1 d-group (mean = 0.87, SEM = 0.027, Welch Two Sample t-test: t(89.89) = 12.95, p = 2.2e-16, Cohen's d = 2.66, n = 47) and again memory performance was enhanced for negative relative to neutral pictures (paired t-test: t(46) = 2.37, p = 0.022, n = 47). Note that for one participant the Free Recall Day2 data was missing. ** p < 0.001.

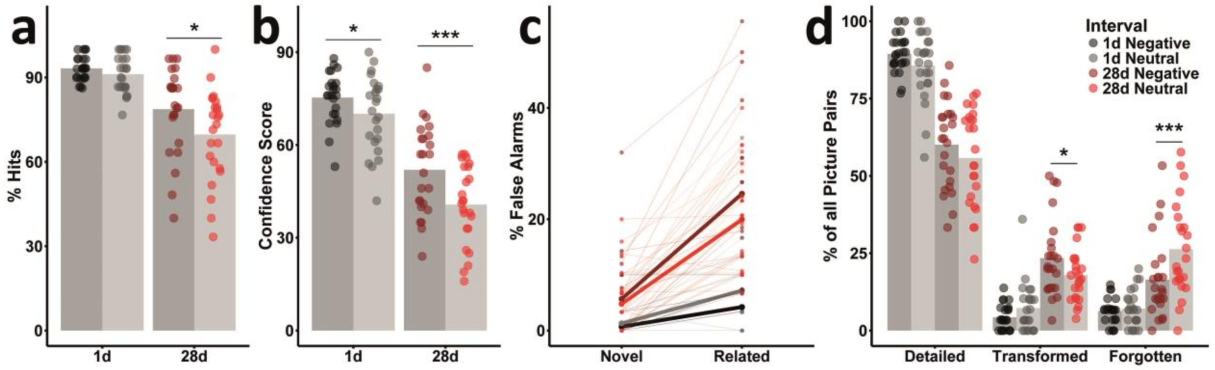

Supplementary Figure 2: Recognition memory for neutral vs. negative pictures. (a) In general, the percentage of hits was higher for negative than for neutral pictures (main effect Emotion: $F(1,46) = 15.44$, $p = 0.0003$, generalized $\eta^2 = 0.054$, $n = 48$), and a Group × Emotion interaction effect ($F(1,46) = 6.089$, $p = 0.0174$, generalized $\eta^2 = 0.022$, $n = 48$) was explained by a significant difference between negative and neutral hits in the 28 d-group (paired t-test: $t(23) = 3.47$, $p = 0.0021$, Cohen's $d = 0.71$, $n = 24$), while there was no significant difference in the 1 d-group (paired t-test: $t(23) = 1.87$, $p = 0.0740$, Cohen's $d = 0.38$, $n = 24$). **(b)** The Confidence Score was higher for negative than for neutral pictures (main effect Emotion: $F(1,46) = 49.85$, $p = 7.41e\text{-}09$, generalized $\eta^2 = 0.107$, $n = 48$), and the difference between negative and neutral pictures was stronger in the 28 d-group than in the 1 d-group (Group × Emotion interaction effect $F(1,46) = 6.59$, $p = 0.0136$, generalized $\eta^2 = 0.016$, $n = 48$; 1 d-group paired t-test: $t(23) = 3.53$, $p = 0.0017$, Cohen's $d = 0.72$, $n = 24$; 28 d-group paired t-test: $t(23) = 6.24$, $p = 2.31e\text{-}06$, Cohen's $d = 1.27$, $n = 24$). **(c)** FA: A significant three-way interaction of Group, Emotion and Picture Type was found ($F(1,46) = 6.62$, $p = 0.0134$, generalized $\eta^2 = 0.011$, $n = 48$; Group × Emotion: $F(1,46) = 6.92$, $p = 0.0116$, generalized $\eta^2 = 0.024$, $n = 48$; main effect Emotion: $F(1,46) = 0.37$, $p = 0.5707$, generalized $\eta^2 = 0.001$, $n = 48$). While there were no significant differences between negative and neutral pictures for FA to novel pictures (Group × Emotion: ($F(1,46) = 1.27$, $p = 0.2662$, generalized $\eta^2 = 0.006$, $n = 48$; 1 d-group paired t-test: $t(23) = -1.51$, $p = 0.195$, Cohen's $d = -0.27$, $n = 24$; 28 d-group paired t-test: $t(23) = 0.72$, $p = 0.48$, Cohen's $d = 0.15$, $n = 24$), there was a significant Group × Emotion interaction for FA to related pictures ($F(1,46) = 8.20$, $p = 0.0063$, generalized $\eta^2 = 0.043$, $n = 48$), with significantly more FA to neutral related pictures compared to negative related pictures in the 1 d-group (paired t-test: $t(23) = -2.07$, $p = 0.05$, Cohen's $d = -0.42$, $n = 24$), while in the 28 d-group there were significantly more FA to negative related pictures compared to neutral related pictures (paired t-test: $t(23) = 2.08$, $p = 0.05$, Cohen's $d = 0.42$, $n = 24$). **(d)** There was no Group × Emotion interaction effect for the detailed pairs ($F(1,46) = 0.02$, $p = 0.8865$, generalized $\eta^2 = 0.0001$, $n = 48$). For transformed pairs there were significantly more negative pairs compared to neutral pairs in the 28 d-group but not the 1 d-group (Group × Emotion interaction: $F(1,46) = 11.20$, $p = 0.0016$, generalized $\eta^2 = 0.057$, $n = 48$; 1 d-group paired t-test: $t(23) = -2.03$, $p = 0.0546$, Cohen's $d = -0.41$, $n = 24$; 28 d-group paired t-test: $t(23) = 2.67$, $p = 0.0138$, Cohen's $d = 0.54$, $n = 24$). For forgotten pairs, on the other hand, there were significantly more neutral pairs compared to negative pairs in the 28 d-group but not the 1 d-group (Group × Emotion interaction: $F(1,46) = 16.09$, $p = 0.0002$, generalized $\eta^2 = 0.045$, $n = 48$; 1 d-group paired t-test: $t(23) = -0.71$, $p = 0.4838$, Cohen's $d = -0.15$, $n = 24$; 28 d-group paired t-test: $t(23) = -5.00$, $p = 4.64e\text{-}05$, Cohen's $d = -1.02$, $n = 24$). ** $p < 0.001$, *** $p < 0.0001$.

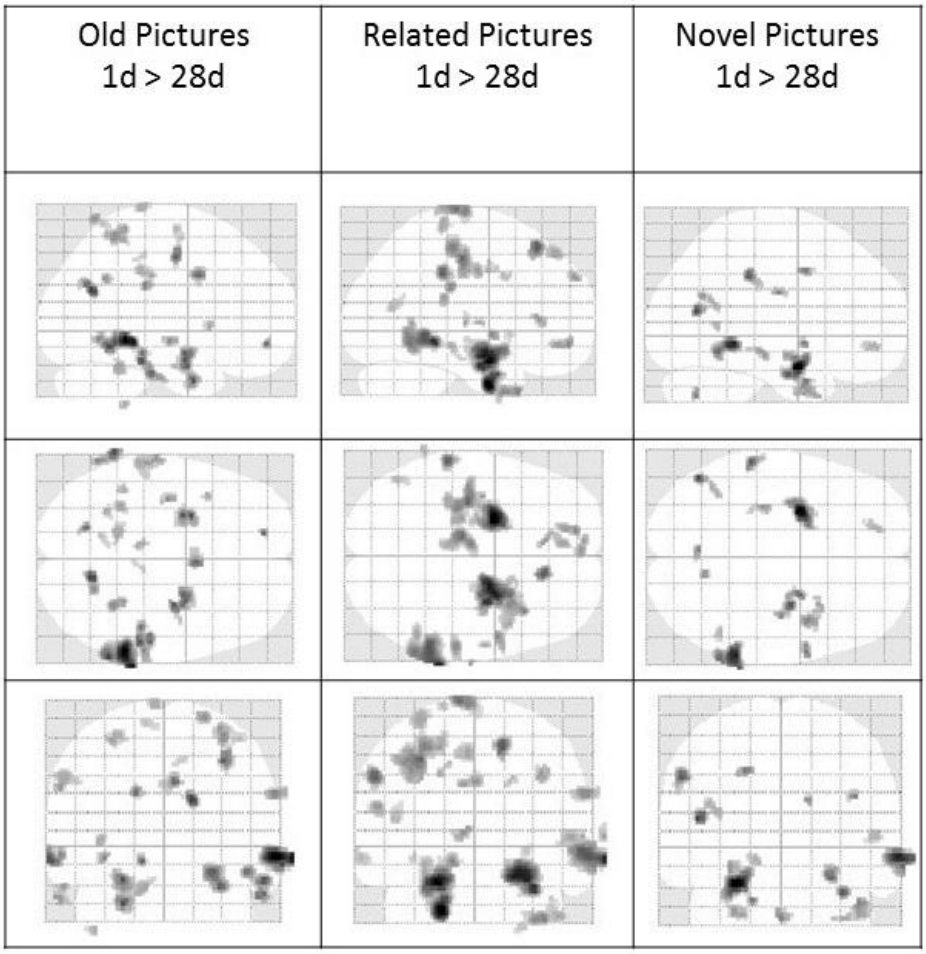

Supplementary Figure 3: Results from the whole brain analysis. Visualizations of the whole brain results: Thresholding: $t > 3.2771$, $p < 0.001$(unc.), df = 46, minimum extent = 10 voxels. Note that there were no significant group differences when a threshold of $p < 0.05$(FWE) was applied. There were also no areas where the 28 d-group showed more activity, even with the more lenient threshold of $p < 0.001$(unc.). Figures were created using SPM. See Supplementary Table 1 for corresponding labels and coordinates.

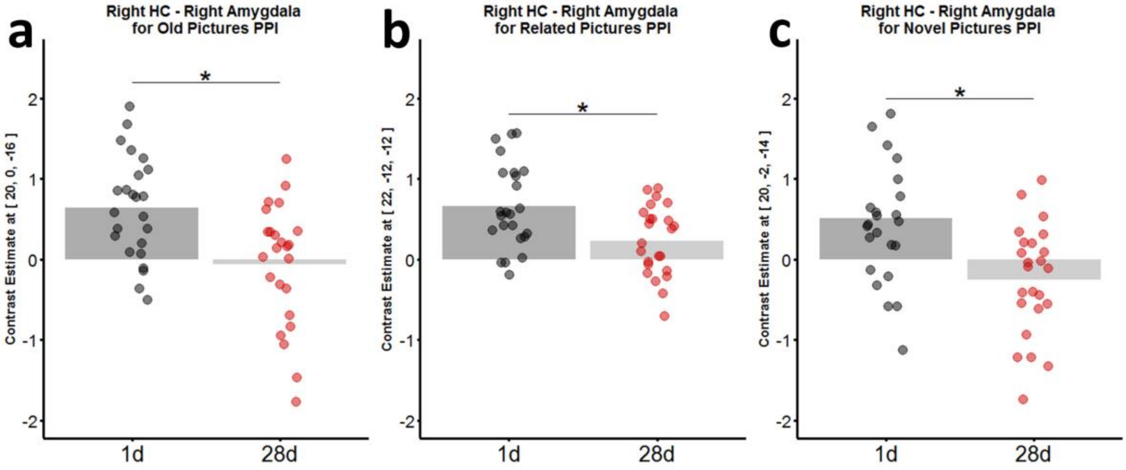

Supplementary Figure 4: Connectivity Right Hippocampus with Right Amygdala. Parameter estimates in the respective peak voxels in the right amygdala. Using the whole hippocampus masks as a seed region in a gPPI analysis showed significant group difference in the connectivity of the right hippocampus to the right amygdala for the main effects of **(a)** old pictures (peak level SVC: x = 20, y = 0, z = -16, t = 3.49, p(FWE) = 0.0187, k = 16), **(b)** related pictures (peak level SVC: x = 22, y = -12, z = -12, t = 3.58, p(FWE) = 0.0161, k = 22) and **(c)** novel pictures (peak level SVC: x = 20, y = -2, z = -14, t = 3.46, p(FWE) = 0.0212, k = 13), with reduced connectivity in the 28 d-group compared to the 1 d-group for all picture types. Neither using the left HC as seed region, nor looking at other ROIs resulted in significant group differences in connectivity. See also Supplementary Table 3. *p < 0.05.

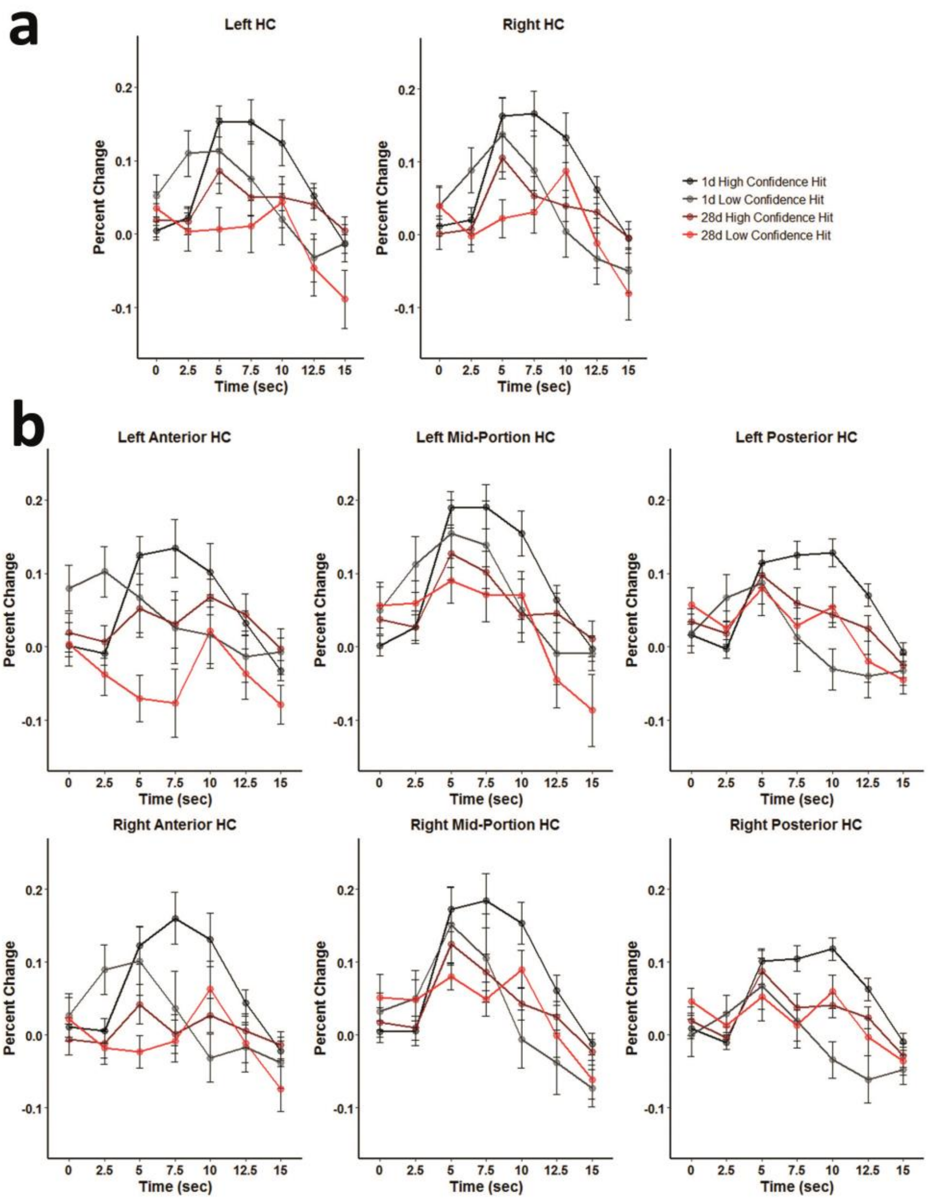

Supplementary Figure 5: High Confidence Hits vs. Low Confidence Hits. (**a**) FIR time courses over the first 15 sec (7TRs) for High Confidence Hits and Low Confidence Hits for each Group (1 d vs 28 d) separately in the left and right hippocampus. Mixed ANOVA models with Group (1 d vs 28 d) and Hit Type (high vs low) were calculated for the peak response (average of the 5s and 7.5s time points). The Group × Hit Type interaction was not significant in these ROIs. The main effect of Hit Type was significant in the left HC ($F(1,46) = 4.66$, $p = 0.036$, generalized $\eta^2 = 0.0367$, $n = 48$), with a trend in the right HC ($F(1,46) = 3.68$, $p = 0.061$, generalized $\eta^2 = 0.0268$, $n = 48$). The main effect of Group was significant in both ROIs (left HC: $F(1,46) = 5.91$, $p = 0.019$, generalized $\eta^2 = 0.0742$, $n = 48$; right HC: $F(1,46) = 5.20$, $p = 0.027$, generalized $\eta^2 = 0.0690$, $n = 48$). Post-hoc Welch Two Sample t-tests comparing the 1 d-group to the 28 d-group were significant for High Confidence Hits (left HC: $t(45.85) = 2.50$, $p = 0.0159$, Cohen's $d = 0.72$, $n = 48$; right HC: $t(45.87) = 2.44$, $p = 0.0188$, Cohen's $d = 0.70$, $n = 48$), but not for Low Confidence Hits (left HC: $t(40.61) = 1.62$, $p = 0.1131$, Cohen's $d = 0.47$, $n = 48$; right HC: $t(34.35) = 1.55$, $p = 0.1295$, Cohen's $d = 0.70$, $n = 48$) **(b)** FIR time courses over the first 15 sec (7TRs) for High Confidence Hits and Low Confidence Hits for each Group (1 d vs 28 d) separately in the hippocampal long axis ROIs. Mixed ANOVA models with Group (1 d vs 28 d) and Hit Type (high vs low) were calculated for the peak response (average of the 5s and 7.5s time points). The Group × Hit Type interaction was not significant in any these ROIs. The main effect of Hit Type was significant in the aHC (left: $F(1,46) = 11.53$, $p = 0.0014$, generalized $\eta^2 = 0.0756$, $n = 48$; right: $F(1,46) = 4.40$, $p = 0.0414$, generalized $\eta^2 = 0.0326$, $n = 48$) and the pHC (left: $F(1,46) = 5.14$, $p = 0.0281$, generalized $\eta^2 = 0.0335$, $n = 48$; right: $F(1,46) = 5.08$, $p = 0.0289$, generalized $\eta^2 = 0.0357$, $n = 48$). The main effect of Group was

only significant in the aHC (left: $F(1,46) = 6.12$, $p = 0.0170$, generalized $\eta^2 = 0.0823$, $n = 48$; right: $F(1,46) = 8.32$, $p = 0.0059$, generalized $\eta^2 = 0.1050$, $n = 48$). Post-hoc Welch Two Sample t-tests comparing the 1 d-group to the 28 d-group were significant for High Confidence Hits (left aHC: $t(45.94) = 2.15$, $p = 0.0369$, Cohen's $d = 0.620$, $n = 48$; right aHC: $t(44.88) = 3.32$, $p = 0.0018$, Cohen's $d = 0.96$, $n = 48$), but not for Low Confidence Hits (left aHC: $t(43.50) = 2.01$, $p = 0.051$, Cohen's $d = 0.58$, $n = 48$; right aHC: $t(34.25) = 1.67$, $p = 0.1044$, Cohen's $d = 0.48$, $n = 48$). Note, however, when correcting for multiple comparisons by the number of ROIs ($0.05/6 = 0.0083$), only the main effect of Hit Type in the left aHC, and the main effect of Group in the right aHC survive this rather conservative correction.

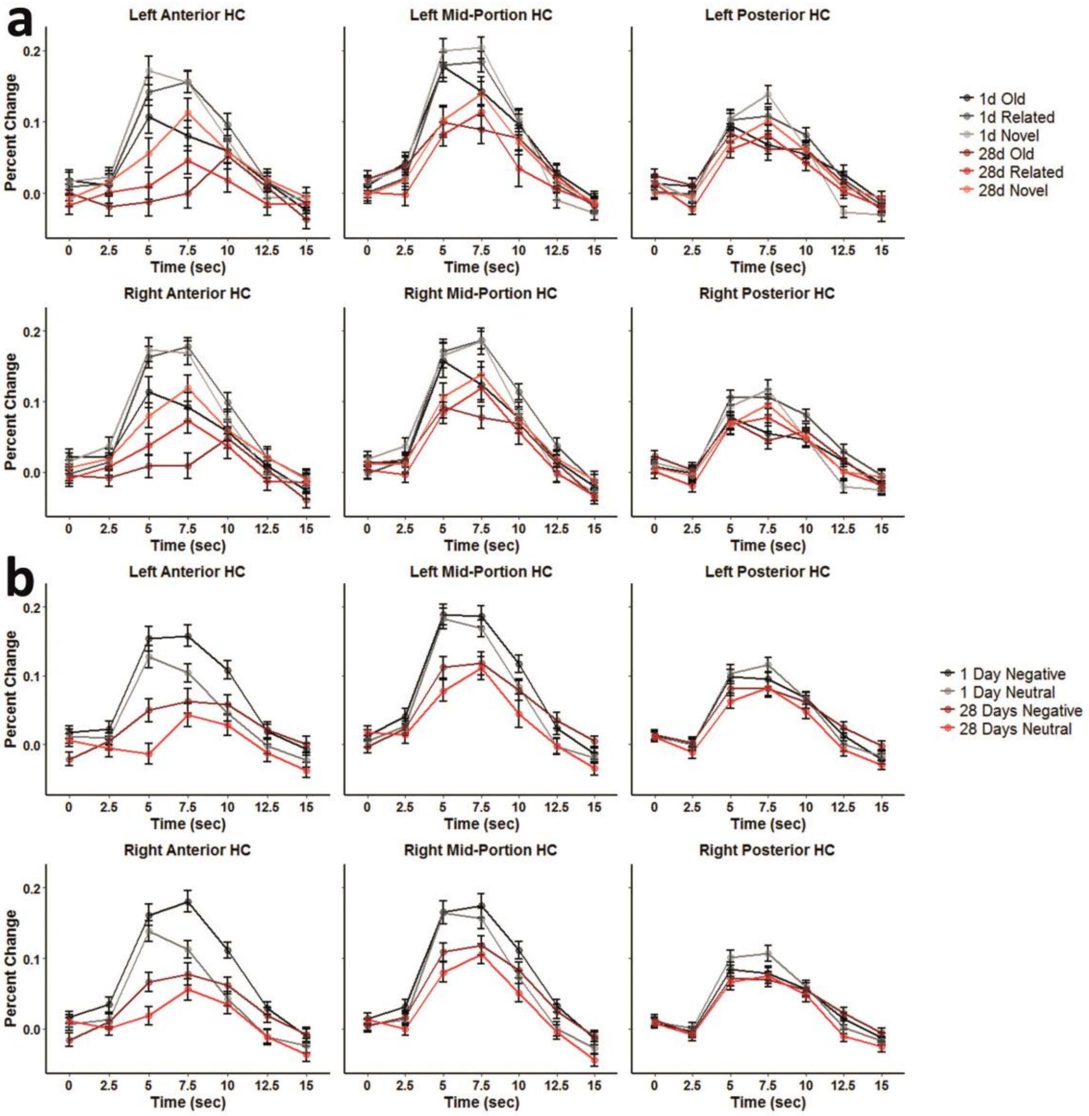

Supplementary Figure 6: Effects of Picture Type and Emotion (**a**) FIR time courses over the first 15 sec (7TRs) for each Picture Type and Group separately. Mixed ANOVA models with Group (1 d vs 28 d), Picture Type (old vs related vs novel) and Emotion (negative vs neutral) were calculated for the peak response (average of the 5s and 7.5s time points). The Group × Picture Type interaction was not significant in any of the ROIs. The main effect of Picture Type was significant in the aHC (left: $F(2,92) = 17.42$, $p = 2.82e-06$, generalized $\eta^2 = 0.0721$, $n = 48$; right: $F(2,92) = 25.90$, $p = 1.09e-07$, generalized $\eta^2 = 0.0957$, $n = 48$), mHC (left: $F(2,92) = 3.97$, $p = 0.0222$, generalized $\eta^2 = 0.0146$, $n = 48$; right: $F(2,92) = 4.82$, $p = 0.010$, generalized $\eta^2 = 0.0173$, $n = 48$), and pHC (left: $F(2,92) = 4.83$, $p = 0.010$, generalized $\eta^2 = 0.0168$, $n = 48$; right: $F(2,92) = 8.77$, $p = 0.0003$, generalized $\eta^2 = 0.0276$, $n = 48$). The time courses show a higher activity increase for novel and related pictures than for old pictures. (**b**) FIR time courses over the first 15 sec (7TRs) for each Emotion and Group separately. Mixed ANOVA models with Group (1 d vs 28 d), Picture Type (old vs related vs novel) and Emotion (negative vs neutral) were calculated for the peak response (average of the 5s and 7.5s time points). The Group × Emotion interaction was not significant in any of the ROIs. The main effect of Emotion was significant in the aHC (left: $F(1,46) = 15.22$, $p = 0.0003$, generalized $\eta^2 = 0.0295$, $n = 48$; right: $F(1,46) = 22.92$, $p = 1.79e-05$, generalized $\eta^2 = 0.0365$, $n = 48$) but not in the mHC or pHC. The activity for negative pictures was higher than for neutral pictures in the aHC.

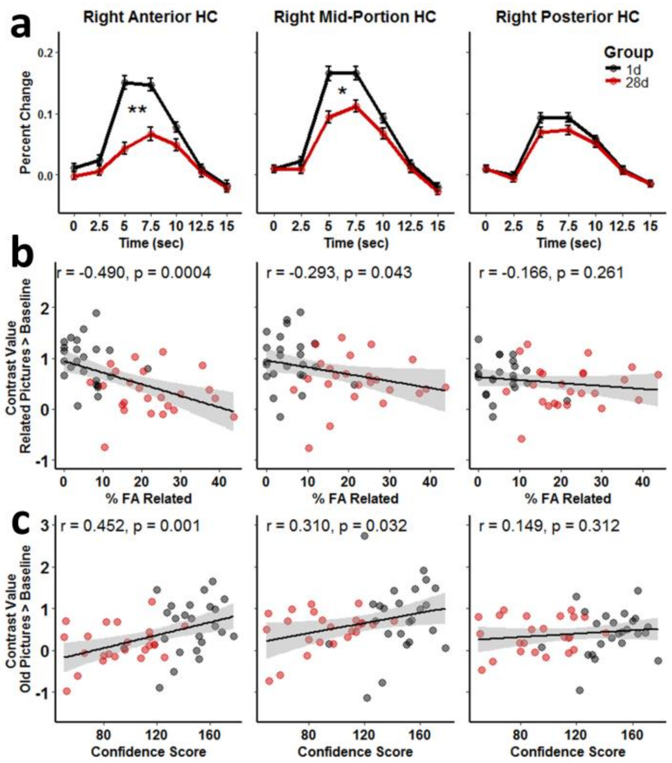

Supplementary Figure 7: Univariate analysis of the right HC long axis ROIs. (**a**) FIR time courses over the first 15 sec (7TRs) for all picture types combined in the right HC long axis ROIs. Mixed ANOVA models with Group (1 d vs 28 d), Picture Type (old vs related vs novel) and Emotion (negative vs neutral) were calculated for the peak response (average of the 5s and 7.5s time points). The activity in the 28 d-group compared to the 1 d-group decreased in the aHC (main effect Group: $F(1,46) = 15.78$, $p = 0.0002$, generalized $\eta^2 = 0.171$, $n = 48$) and mHC ($F(1,46) = 5.05$, $p = 0.029$, generalized $\eta^2 = 0.067$, $n = 48$). There was no difference between the groups in the pHC ($F(1,46) = 1.24$, $p = 0.27$, generalized $\eta^2 = 0.017$, $n = 48$). A Group × HC Long Axis interaction was significant ($F(2,92) = 6.16$, $p = 0.0062$, generalized $\eta^2 = 0.032$, $n = 48$) (**b**) Correlations of the percentage of related FA with the contrast value for related pictures > baseline. There was a negative correlation in the aHC ($t(46) = -3.82$, $p = 0.0004$, $r = -0.49$, $n = 48$) when calculating across all participants, however this correlation was not significant when looking at each group separately (1 d-group: $r = -0.23$, $p = 0.29$, $n = 24$; 28 d-group: $r = -0.08$, $p = 0.71$, $n = 24$). In the mHC there was also a significant correlation when looking at the whole group ($t(46) = -2.08$, $p = 0.0431$, $r = -0.29$, $n = 48$) but not in each group separately (1 d-group: $r = -0.09$, $p = 0.68$, $n = 24$; 28 d-group: $r = 0.02$, $p = 0.93$, $n = 24$). In the pHC neither the correlation across all participants ($t(46) = -1.14$, $p = 0.2607$, $r = -0.17$, $n = 48$) nor in each group separately was significant (1 d-group: $r = -0.15$, $p = 0.49$, $n = 24$; 28 d-group: $r = 0.08$, $p = 0.71$, $n = 24$). The correlations across all participants were significantly distinct in the right aHC and pHC (Pearson and Filon's $z = -2.18$, $p = 0.0291$, $n = 48$). (**c**) Correlations of the Confidence Score with the contrast value for old pictures > baseline. There was a positive correlation in the right aHC across all subjects ($t(46) = 3.44$, $p = 0.0013$, $r = 0.45$, $n = 48$), but not for each group separately (1 d-group: $r = 0.28$, $p = 0.19$, $n = 24$; 28 d-group: $r = 0.30$, $p = 0.15$, $n = 24$). In the mHC the correlation was significant across all participants ($t(46) = 2.21$, $p = 0.0320$, $r = 0.31$, $n = 48$), but not in each group separately (1 d-group: $r = 0.18$, $p = 0.41$, $n = 24$; 28 d-group: $r = 0.33$, $p = 0.12$, $n = 24$). In the pHC neither the correlation across all participants ($t(46) = 1.02$, $p = 0.3118$, $r = 0.15$, $n = 48$), nor in each group separately was significant (1 d-group: $r = 0.23$, $p = 0.29$, $n = 24$; 28 d-group: $r = 0.15$, $p = 0.49$, $n = 24$). The correlations across all participants were significantly distinct in the right aHC and pHC (Pearson and Filon's $z = 2.22$, $p = 0.0266$, $n = 48$). These data therefore mainly replicate the findings of the left hippocampus shown in Figure 2, just that the correlations in each group separately for the percentage of related FA are not significant in the right aHC, in comparison to the left aHC. * $p < 0.05$, ** $p < 0.001$, all error bars are SEM.

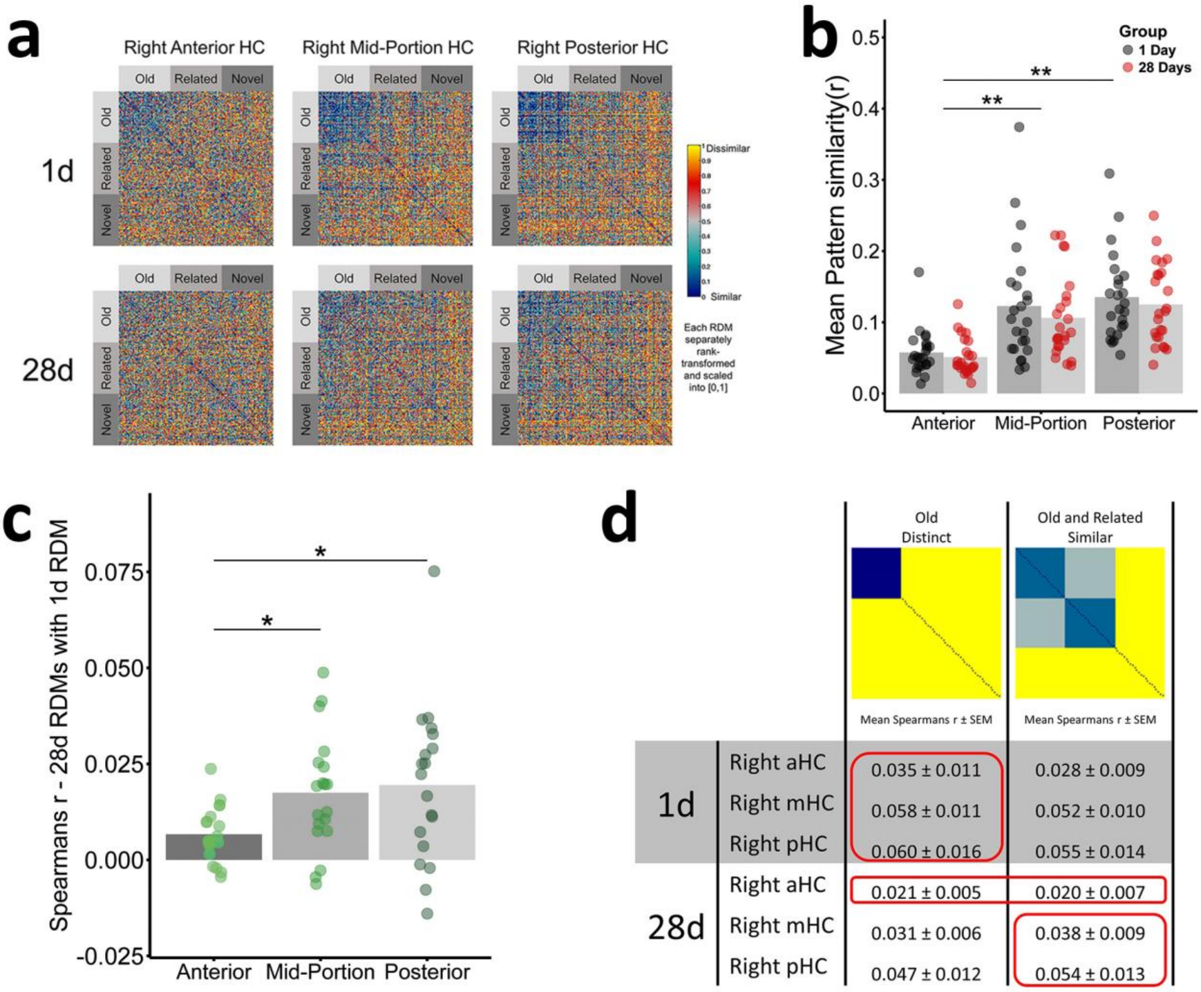

Supplementary Figure 8: Representational Similarity Analysis (RSA) of the right HC long axis ROIs. (**a**) The group average RDMs of the right long axis hippocampal ROIs (1 d-group in the first row, 28 d-group in the second row). Blue colors = most similar, bright colors = most dissimilar; note that for the visualizations each RDM was separately rank transformed and scaled into [0, 1]. These data are from only 40 participants (1 d-group = 21, 28 d-group = 19) as the remaining 8 participants had different sized RDMs and could therefore not be included in the average RDM for visualization. (**b**) Comparison of mean pattern similarities (Pearsons r) across ROIs. The mean similarity in the hippocampal subregions differed significantly (main effect HC Long Axis: $F(2,92) = 54.00$, $p = 3.07e-16$, generalized $\eta^2 = 0.264$, $n = 48$) , yet without group differences (main effect Group: $F(1,46) = 0.70$, $p = 0.4084$, generalized $\eta^2 = 0.010$, $n = 48$; interaction effect Group × HC Long Axis : $F(2,92) = 0.26$, $p = 0.7982$, generalized $\eta^2 = 0.0015$, $n = 48$). (**c**) Comparison between Groups: correlations (Spearmans r) of each single subject RDM of the 28 d-group to the respective average RDM of the 1 d-group. The correlations between the two groups differed significantly in the three ROIs (main effect HC Long Axis: $F(2,36) = 5.64$, $p = 0.0074$, generalized $\eta^2 = 0.1249$, $n = 19$). Note that these data are from only 40 participants (1 d-group = 21, 28 d-group = 19) as the remaining 8 participants had different sized RDMs and could therefore not be included in the average RDM for the group comparisons. (**d**) Comparison with two model RDMs (Online Methods): each cell in the table shows the mean of the correlations (Spearmans r ± SEM) of the single-subject brain RDMs with the respective model RDM (first three rows = 1 d-group, last three rows = 28 d-group; note that all n = 48 participants are included here). For each ROI the model with the higher correlation was marked by a red frame, in case of very similar correlations both values were marked. In the 1 d-group, the “Old Distinct” model had a trend towards a better fit in the right aHC (one-tailed paired t-test: $t(23) = 1.15$, $p = 0.1301$, $n = 24$) and right mHC (one-tailed paired t-test: $t(23) = 1.19$, $p = 0.1230$, $n = 24$), but less so in the right pHC (one-tailed paired t-test: $t(23) = 0.77$, $p = 0.2243$, $n = 24$). In the 28 d-group the “Old and Related Similar” model showed a trend towards a

better fit in the right mHC (one-tailed paired t-test: $t(23) = -1.08$, $p = 0.1447$, $n = 24$) and in the right pHC (one-tailed paired t-test: $t(23) = -1.16$, $p = 0.1403$, $n = 24$), while in the right aHC both models were indistinguishable (one-tailed paired t-test: $t(23) = 0.25$, $p = 0.5968$, $n = 24$) and the model fits were generally rather low. These data therefore mainly replicate the findings of the left hippocampus.* $p < 0.05$, ** $p < 0.001$.

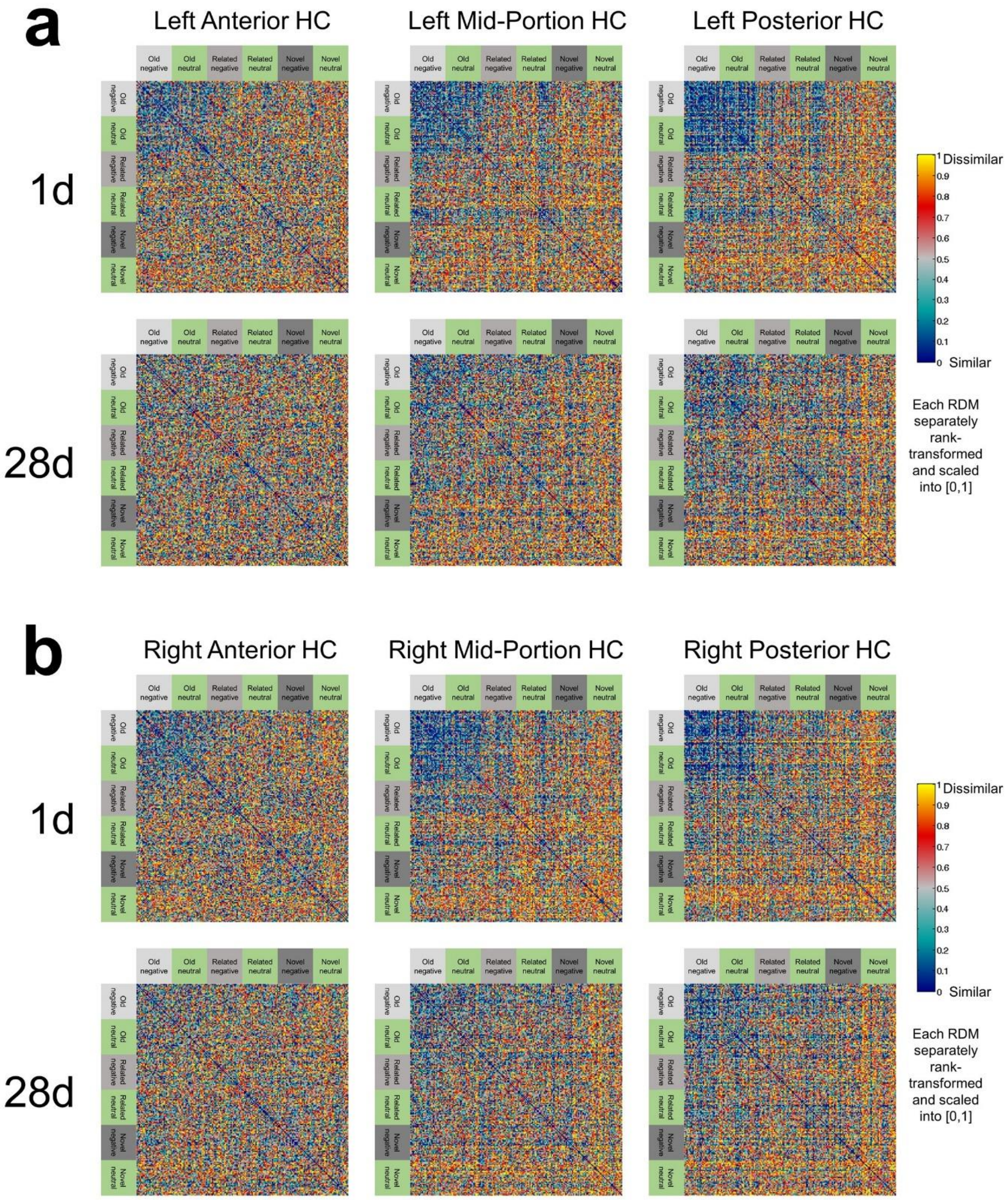

Supplementary Figure 9: Looking at Emotion in the RDMs. (**a**) The group average RDMs of the left long axis hippocampal ROIs with indications of the picture emotionality. (1 d-group in the first row, 28 d-group in the second row). (**b**) The group average RDMs of the right long axis hippocampal ROIs with indications of the picture emotionality. (1 d-group in the first row, 28 d-group in the second row). Blue colors = most similar, bright colors = most dissimilar. Note that for the visualizations each RDM was separately rank transformed and scaled into [0, 1]. Note that these data are from only 40 participants (1 d-group = 21, 28 d-group = 19) as the remaining 8 participants had different sized RDMs and could therefore not be included in the average RDM for visualization.

Supplementary Table 1. Results from the whole brain analysis.

	Region Label	Extent	t-value	x	y	z
**Old Pictures**	Middle Temporal Gyrus, temporooccipital part	350	5.00	62	-44	-8
**1 d > 28 d**	Precuneous Cortex	37	4.43	14	-66	24
	Parahippocampal Gyrus, anterior division	186	4.32	-26	2	-34
	Inferior Temporal Gyrus, posterior division	118	4.30	56	-32	-20
	Inferior Temporal Gyrus, temporooccipital part	90	4.29	-68	-50	-10
	Right Cerebral White Matter	66	4.21	32	-10	46
	Right Amygdala	135	4.20	26	-4	-16
	Cingulate Gyrus, anterior division	72	4.15	4	6	34
	Left Cerebral White Matter	15	4.14	-14	50	-10
	Precuneous Cortex	24	4.09	-18	-68	28
	Supramarginal Gyrus, anterior division	36	3.93	62	-30	28
	Left Cerebral White Matter	32	3.90	-36	-14	-8
	Superior Parietal Lobule	66	3.88	32	-50	58
	Lateral Occipital Cortex, superior division	21	3.84	22	-64	70
	Angular Gyrus	17	3.82	-62	-58	32
	Inferior Temporal Gyrus, posterior division	50	3.78	-60	-28	-24
	Superior Parietal Lobule	43	3.75	-32	-46	58
	*Cerebelum_4_5_L	38	3.70	-12	-48	-24
	Supramarginal Gyrus, anterior division	74	3.68	-60	-26	34
	Postcentral Gyrus	25	3.66	-6	-34	78
	Insular Cortex	16	3.65	-38	12	2
	*Cerebelum_8_L	10	3.63	-42	-44	-48
	Lateral Occipital Cortex, superior division	19	3.50	-12	-60	68
	Cingulate Gyrus, posterior division	10	3.43	8	-32	46
**Related Pictures**	Parahippocampal Gyrus, anterior division	713	5.658	-20	-2	-38
**1 d > 28 d**	Left Hippocampus	713	3.799	-30	-12	-20
	Right Amygdala	598	5.286	22	-6	-18
	Right Putamen	598	3.547	28	12	-4
	Middle Temporal Gyrus, temporooccipital part	551	5.118	72	-40	-8
	Middle Temporal Gyrus, temporooccipital part	551	3.879	54	-54	4
	Superior Frontal Gyrus	75	4.634	14	30	52
	Supramarginal Gyrus, anterior division	89	4.559	-58	-34	36
	Left Cerebral White Matter	433	4.524	-32	-20	46
	Precentral Gyrus	191	4.434	-10	-24	78
	Temporal Pole	183	4.275	38	18	-42
	Right Cerebral White Matter	183	3.502	42	2	-30
	Temporal Pole	183	3.389	22	6	-48
	Left Cerebral White Matter	53	4.162	-14	52	-10
	Planum Polare	35	4.117	54	0	-4
	Supramarginal Gyrus, anterior division	50	4.116	60	-28	28
	Left Cerebral White Matter	19	4.002	-8	32	6
	Superior Frontal Gyrus	49	3.997	-2	56	34
	Parietal Operculum Cortex	37	3.995	-58	-32	20
	Right Cerebral White Matter	33	3.968	36	-10	38
	Frontal Pole	52	3.890	-6	42	50
	Superior Temporal Gyrus, posterior division	11	3.881	72	-18	4
	Postcentral Gyrus	53	3.856	-32	-32	70
	Left Hippocampus	26	3.818	-30	-28	-12
	Left Cerebral White Matter	15	3.803	-22	-2	38
	Planum Temporale	11	3.803	60	-8	4

	Lateral Occipital Cortex, inferior division	32	3.685	-46	-68	14
	Middle Temporal Gyrus, temporooccipital part	22	3.656	-66	-50	-8
	Right Cerebral White Matter	12	3.623	18	8	34
	Precentral Gyrus	11	3.609	-20	-16	76
	Left Cerebral White Matter	10	3.492	-36	-14	-8
**Novel Pictures**	Left Amygdala	210	4.727	-28	-2	-22
**1 d > 28 d**	Middle Temporal Gyrus, temporooccipital part	181	4.450	64	-44	-6
	Lateral Occipital Cortex, inferior division	35	4.203	-48	-68	14
	Supramarginal Gyrus, anterior division	59	4.086	-58	-34	36
	Right Hippocampus	26	4.067	32	-8	-26
	Right Amygdala	42	3.968	26	-2	-18
	Left Cerebral White Matter	19	3.884	-22	4	40
	Superior Temporal Gyrus, anterior division	21	3.867	62	2	-6
	*Vermis_8	20	3.851	-2	-68	-40
	Right Cerebral White Matter	67	3.814	42	4	-32
	Right Cerebral White Matter	15	3.811	40	-14	26
	Precuneous Cortex	10	3.716	12	-64	24
	Temporal Pole	42	3.701	-28	4	-36
	Left Hippocampus	26	3.701	-30	-24	-14
	Left Cerebral White Matter	25	3.579	-16	48	-8
	Angular Gyrus	24	3.571	-42	-60	22
	Middle Temporal Gyrus, temporooccipital part	18	3.551	50	-54	4

Anatomical labels and coordinates of the whole brain results: thresholding: $t > 3.2771$, $p < 0.001$(unc.), df = 46, minimum extent = 10 voxels. Table shows all local maxima separated by more than 20 mm. Regions were automatically labeled using the Harvard Oxford Atlas (for locations not found in the Harvard Oxford Atlas, we used the AAL2 atlas for labeling, these regions are marked by a *****). x, y, and z =Montreal Neurological Institute (MNI) coordinates in the left-right, anterior-posterior, and inferior-superior dimensions, respectively. Note that there were no significant group differences when a threshold of $p < 0.05$(FWE) was applied. There were also no areas where the 28 d-group showed more activity, even with the more lenient threshold of $p < 0.001$(unc.). Tables were created using bspmview (http://www.bobspunt.com/bspmview/). See Supplementary Figure 3 for corresponding visualizations.

Supplementary Table 2. Statistical comparisons on the peak responses of the FIR time courses for several memory-related ROIs.

Effect	Region	$F_{(1,46)}$	p	Generalized η^2
Main Effect **Group**	Left hippocampus	14.49	**0.0004***	0.1486
	Right hippocampus	8.99	0.0044	0.1052
	Left anterior parahippocampal gyrus	14.64	**0.0004***	0.1544
	Right anterior parahippocampal gyrus	9.65	0.0032	0.0962
	Left posterior parahippocampal gyrus	9.28	0.0038	0.1353
	Right posterior parahippocampal gyrus	6.70	0.0128	0.1018
	Left Amygdala	14.87	**0.0004***	0.1720
	Right Amygdala	17.97	**0.0001***	0.1868
	Precuneous Cortex	1.17	0.2848	0.0104
	Left angular gyrus	6.48	0.0143	0.0810
	Right angular gyrus	1.01	0.3199	0.0159
	Anterior cingulate gyrus	9.76	0.0031	0.0677
	Left inferior frontal gyrus, pars opercularis	2.59	0.1142	0.0299
	Right inferior frontal gyrus, pars opercularis	0.03	0.8624	0.0003
	Left inferior frontal gyrus, pars triangularis	0.64	0.4272	0.0078
	Right inferior frontal gyrus, pars triangularis	7.08	0.0107	0.0888
	Left temporal pole	5.44	0.0241	0.0494
	Right temporal pole	12.04	**0.0011***	0.0804
		$F_{(2,92)}$	**p**	**Generalized η^2**
Main Effect **Picture Type**	Left hippocampus	9.52	**0.0002***	0.0367
	Right hippocampus	11.61	**<0.0001****	0.0438
	Left anterior parahippocampal gyrus	0.12	0.8907	0.0005
	Right anterior parahippocampal gyrus	0.07	0.9361	0.0003
	Left posterior parahippocampal gyrus	0.74	0.4815	0.0014
	Right posterior parahippocampal gyrus	0.25	0.7824	0.0005
	Left Amygdala	16.74	**<0.0001****	0.0490
	Right Amygdala	16.75	**<0.0001****	0.0530
	Precuneous Cortex	1.95	0.1481	0.0102

	Left angular gyrus	0.59	0.5557	0.0020
	Right angular gyrus	4.00	0.0216	0.0118
	Anterior cingulate gyrus	8.16	**0.0005***	0.0564
	Left inferior frontal gyrus, pars opercularis	19.72	**<0.0001****	0.0886
	Right inferior frontal gyrus, pars opercularis	10.00	**0.0001***	0.0475
	Left inferior frontal gyrus, pars triangularis	1.70	0.1890	0.0072
	Right inferior frontal gyrus, pars triangularis	1.93	0.1509	0.0077
	Left temporal pole	2.33	0.1027	0.0112
	Right temporal pole	2.98	0.0556	0.0156
		**$F_{(1,46)}$**	p	**Generalized η^2**
Main Effect **Emotion**	Left hippocampus	1.53	0.2230	0.0028
	Right hippocampus	1.36	0.2499	0.0019
	Left anterior parahippocampal gyrus	5.99	0.0182	0.0123
	Right anterior parahippocampal gyrus	3.71	0.0602	0.0065
	Left posterior parahippocampal gyrus	16.84	**0.0002***	0.0169
	Right posterior parahippocampal gyrus	28.26	**<0.0001****	0.0247
	Left Amygdala	54.40	**<0.0001****	0.0798
	Right Amygdala	51.65	**<0.0001****	0.0944
	Precuneous Cortex	0.27	0.6031	0.0007
	Left angular gyrus	0.01	0.9401	0.00001
	Right angular gyrus	5.58	0.0225	0.0044
	Anterior cingulate gyrus	0.94	0.3368	0.0025
	Left inferior frontal gyrus, pars opercularis	0.33	0.5662	0.0007
	Right inferior frontal gyrus, pars opercularis	0.92	0.3428	0.0020
	Left inferior frontal gyrus, pars triangularis	0.99	0.3253	0.0025
	Right inferior frontal gyrus, pars triangularis	10.54	**0.0021***	0.0144
	Left temporal pole	10.27	**0.0025***	0.0266
	Right temporal pole	8.85	0.0046	0.0284

A mixed ANOVA model was calculated with Group (1 d vs 28 d) as between-subject factor and Picture Type (old vs related vs novel) and Emotion (negative vs neutral) as within-subject factors. The main effects are listed in the table. Note that none of the interactions **(Group × Picture Type × Emotion; Group × Picture Type; Group × Emotion and Picture Type × Emotion)** were significant in any of the ROIs. For a definition of the peak responses in each ROI see methods. Effects are marked as significant when falling beneath p-value thresholds corrected for the number of ROIs (*****0.05 / 18 ROIs = 0.0027; ******0.001/18 ROIs = 0.0001).

Supplementary Table 3. Results from the gPPI analysis

Seed Region	Contrast	Group Comparison	ROI	Extent k	p-value	t-value	x	y	z
**Left HC**	PPI Old Negative > PPI Old Neutral	28 d > 1 d	Right inferior frontal gyrus, pars opercularis	11	0.006	3.84	52	18	6
	PPI Old Negative > PPI Old Neutral	28 d > 1 d	Right Amygdala	20	0.003	4.17	24	-2	-14
	PPI Old Negative > PPI Old Neutral	28 d > 1 d	Right inferior frontal gyrus, pars opercularis	28	0.011	3.64	52	16	24
**Right HC**	PPI Novel	1 d > 28 d	Right Amygdala	13	0.021	3.45	20	-2	-14
	PPI Old	1 d > 28 d	Right Amygdala	16	0.018	3.49	20	0	-16
	PPI Related	1 d > 28 d	Right Amygdala	22	0.016	3.57	22	-12	-12
	PPI Old Negative > PPI Old Neutral	28 d > 1 d	Left inferior frontal gyrus, pars opercularis	16	0.007	3.93	-56	20	16
	PPI Old Negative > PPI Old Neutral	28 d > 1 d	Right Temporal Pole	19	0.014	4.24	56	8	-6
	PPI Old Negative > PPI Old Neutral	28 d > 1 d	Right inferior frontal gyrus, pars opercularis	42	0.004	3.97	52	14	22
**Left aHC**	PPI Old Negative > PPI Old Neutral	28 d > 1 d	Left temporal pole	11	0.017	4.17	-48	12	-26
**Right aHC**	PPI Related	1 d > 28 d	Right Amygdala	15	0.015	3.58	20	-4	-14
	PPI Old Negative > PPI Old Neutral	28 d > 1 d	Left temporal pole	10	0.017	4.18	-58	8	-6
	PPI Old Negative > PPI Old Neutral	28 d > 1 d	Left inferior frontal gyrus, pars triangularis	24	0.004	3.95	-52	26	8
	PPI Old Negative > PPI Old Neutral	28 d > 1 d	Right temporal pole	44	0.008	4.45	56	8	-6
	PPI Novel Negative > PPI Novel Neutral	28 d > 1 d	Anterior cingulate gyrus	80	0.010	4.39	-4	12	34
	PPI Old Negative > PPI Old Neutral	28 d > 1 d	Left inferior frontal gyrus, pars opercularis	106	0.001	4.44	-54	12	0

We used the Left HC, Right HC, Left aHC, Right aHC, Left pHC, Right pHC as seed regions. We tested the main effects for *PPI Old*, *PPI Related* and *PPI Novel,* and the differences contrast *PPI Old Negative > PPI Old Neutral, PPI Related Negative > PPI Related Neutral, PPI Novel Negative > PPI Novel Neutral* with two-sample t-tests, comparing the 1 d-group and 28 d-group. We applied a small volume correction (SVC) for all our ROIs (see Supplementary Table 2 for all included ROIs) to find areas which show a significant difference in their connectivity to the seed region between the two groups. Voxels were regarded as significant when falling below a corrected voxel threshold of 0.05 (FWE) adjusted for the small volume. All areas with k > 10 significant voxels are reported.
